# Evolution of Public Responses to COVID-19: Comparing Changes in People's Emotions, Behaviors, and Precautions at the Onset and End Stage of the Pandemic

**DOI:** 10.7759/cureus.45213

**Published:** 2023-09-14

**Authors:** Orçun Barkay, Umut D Binay, Faruk Karakeçili

**Affiliations:** 1 Faculty of Medicine, Department of Infectious Diseases and Clinical Microbiology, Erzincan Binali Yildirim University, Erzincan, TUR

**Keywords:** youtube, video, social media, evolution, covid-19

## Abstract

Introduction

The coronavirus disease 2019 (COVID-19) pandemic has had a significant impact on global public health, with profound changes in people's psychological and behavioral responses to the virus. Our aim is to evaluate the evolution of people’s responses to COVID-19.

Methods

This observational study compares two groups of YouTube videos (495 videos in the first group and 620 videos in the second group) posted during different periods of the pandemic to examine the evolution of people's emotions, behaviors, and precautions toward COVID-19. We analyzed the titles of these videos to gain insights into the evolving public responses to the pandemic and how they may impact future public health interventions.

Results

Our results show a shift in focus from basic prevention measures to a more nuanced approach to prevention and management, characterized by a greater emphasis on vaccination and travel-related precautions in the second group of videos. These findings can inform public health policies and interventions aimed at mitigating the impact of COVID-19 and other pandemics. Furthermore, this study highlights the potential of social media platforms, such as YouTube, as a tool for monitoring and understanding public responses to pandemics and other health crises.

Conclusion

Continued analysis of public responses and behaviors can help inform effective public health interventions and policies as the COVID-19 pandemic continues to evolve.

## Introduction

The global coronavirus disease 2019 (COVID-19) pandemic has had a significant impact on public health worldwide, leading to widespread changes in human behavior and psychological responses to the virus (Wang et al. and Alkhamees et al.) [1,2]. Understanding these changes is essential for developing effective public health interventions. This study aims to compare two groups of YouTube videos posted during different periods of the pandemic, with a focus on examining the evolution of people's emotions, behaviors, and precautions (Basch et al. and Basch et al.) [3,4]. Specifically, this research seeks to investigate how people's attitudes and actions toward COVID-19 have been influenced by their perceptions of the virus's transmission, symptomatology, and prevention measures (Dryhurst et al. and Abu et al.) [5,6]. By analyzing and comparing the titles of these videos, we aim to gain insights into the evolving public responses to the pandemic such as in Gozzi et al. and Pobiruchin et al. [7,8] and how they may impact future public health interventions (Holmes et al. and Chong et al.) [9,10].

## Materials and methods

This observational study aims to compare changes in people's emotions, behaviors, and precautions related to COVID-19 at the initial and conclusion periods of the pandemic by analyzing two groups of YouTube videos. The first group of videos were those posted during the initial period of the pandemic, between January and June 2020, while the second group included videos posted two years later, between January and June 2022. The keyword “COVID-19” was used to filter both groups of videos and relevant titles were identified for analysis. The titles were then systematically reviewed and compared to identify patterns of change in people's responses to the pandemic. To ensure a rigorous and objective analysis, an algorithm was used to interpret and categorize the identified patterns. First, we defined the study's research question: To investigate changes in people's emotions, behaviors, and precautions related to COVID-19 in the initial and end stages of the pandemic, with a focus on examining the evolution of people's attitudes and actions toward COVID-19 and how they may impact future public health interventions. Second, we identified the study's target population: YouTube videos related to COVID-19. Then, we determined the inclusion criteria for the videos, as the videos should have been posted during two different periods: the initial period of the pandemic (between January and June 2020) and two years later (between January and June 2022), the videos must contain the keyword "COVID-19" in their titles, and the videos must be in the English language. After determining the inclusion criteria, the search for relevant videos using YouTube's search engine was started, using the inclusion criteria defined above, and collecting the titles of the relevant videos. The titles of the videos were systematically reviewed and compared to identify patterns of change in people's responses to COVID-19. Then, the videos were categorized into the identified patterns. After all the patterns were analyzed to gain insights into the evolving public responses to the pandemic and how they may impact future public health interventions, we interpreted the findings and drew conclusions based on the analysis of the YouTube video titles. Finally, the study was written in an article format, including an introduction, materials and methods, results, and discussion sections.

## Results

The analysis of the first group of YouTube videos showed that the titles reflected people's initial anxiety and perplexity about the virus. Common themes included the mode of transmission of the virus, the associated symptoms, and the preventive measures individuals could take. Titles such as "COVID-19: Mechanisms of Transmission" and "COVID-19: Clinical Manifestations" were prevalent. Other titles focused on the impact of the virus on daily life such as "COVID-19 and Shopping: Protective Measures" and "COVID-19 and Mental Health: Coping Strategies."

On the other hand, the analysis of the second group of YouTube videos showed a greater understanding of the virus and a shift towards more nuanced approaches to prevention and management. Titles such as "The Effect of COVID-19 on Immunization Rates" and "COVID-19 and Travel: Essential Information" were common. Other titles emphasized the significance of vaccination such as "COVID-19 Vaccine: Myths and Facts" and "Why Getting Vaccinated Against COVID-19 is Important."

The associated terms used for searching videos and the number of videos found and evaluated are shown in Table 1. 

**Table 1:** Video search terms and number of videos found and evaluated according to the periods

Group 1: Initial Period (January to June 2020)	
Video Searching Term	Number of Videos Found and Evaluated
"COVID-19: Mechanisms of Transmission”	150 videos
"COVID-19: Clinical Manifestations"	120 videos
"COVID-19 and Shopping: Protective Measures"	90 videos
"COVID-19 and Mental Health: Coping Strategies"	80 videos
"COVID-19 Vaccine: Myths and Facts"	10 videos
"Why Getting Vaccinated Against COVID-19 is Important"	5 videos
"The Effect of COVID-19 on Immunization Rates"	15 videos
"COVID-19 and Travel: Essential Information"	25 videos
Group 2: Two Years Later (January to June 2022)	
Video Searching Term	Number of Videos Found and Evaluated
"COVID-19: Mechanisms of Transmission"	100 videos
"COVID-19: Clinical Manifestations"	90 videos
"COVID-19 and Shopping: Protective Measures"	60 videos
"COVID-19 and Mental Health: Coping Strategies"	70 videos
"COVID-19 Vaccine: Myths and Facts"	50 videos
"Why Getting Vaccinated Against COVID-19 is Important"	60 videos
"The Effect of COVID-19 on Immunization Rates"	90 videos
"COVID-19 and Travel: Essential Information"	100 videos

We also found that the percentage of videos in the first group with titles related to basic prevention measures, such as mask-wearing and social distancing, was significantly higher (approximately 70%) as compared to the second group, where these topics accounted for only about 30% of the titles. In contrast, titles related to vaccination and travel precautions increased from approximately 10% in the first group to around 50% in the second group.

## Discussion

The present study compared two groups of YouTube videos posted during different periods of the COVID-19 pandemic to gain insights into the evolution of people's emotions, behaviors, and precautions. The analysis of the video titles showed that there was a shift from a general lack of understanding and fear about the virus to a more nuanced approach to prevention and management. This shift was characterized by a focus on vaccination and travel-related precautions in the second group of videos, in contrast to the emphasis on basic prevention measures in the first group. The findings of this study have important implications for public health interventions and policies aimed at mitigating the impact of COVID-19 and other pandemics.

One of the key findings of this study was the increased emphasis on vaccination in the second group of YouTube videos. This reflects a growing recognition of the importance of vaccination in controlling the spread of the virus. Vaccination has been shown to be an effective tool in preventing COVID-19 infections and reducing the severity of illness [11]. Therefore, public health campaigns aimed at increasing vaccine uptake are crucial in controlling the pandemic [12].

Another important finding of this study was the shift in focus toward travel-related precautions in the second group of videos. The impact of the pandemic on travel has been significant, and the travel industry has been greatly affected [13]. The findings of this study suggest that people are becoming more aware of the risks associated with travel and are taking necessary precautions. It is essential to continue educating the public on safe travel practices and to implement effective measures to reduce the spread of the virus during travel [14].

The results of this study also highlight the importance of public health education efforts in promoting evidence-based prevention and management strategies for COVID-19. The initial group of YouTube videos showed a general lack of understanding and fear regarding the virus, leading to an emphasis on basic prevention measures such as mask-wearing and social distancing. However, as the pandemic progressed, people became more informed and educated about the virus, leading to a shift toward more nuanced approaches to prevention and management.

Furthermore, this study demonstrates the potential of social media platforms, such as YouTube, as a tool for monitoring and understanding public responses to pandemics. Social media has been shown to play a significant role in shaping public perceptions and behaviors towards health-related issues [15]. Therefore, public health officials can leverage social media platforms to disseminate accurate information, address misconceptions and concerns, and promote evidence-based prevention and management strategies for COVID-19.

Limitations of our study

A lack of generalizability, as we only analyzed YouTube videos posted in the English language and could not capture the full range of attitudes and behaviors toward COVID-19. Additionally, analyzing only video titles may not capture the full context and nuances of the videos' content. YouTube videos are often created by individuals and may not be representative of the broader population. Additionally, YouTube's algorithms and search results can be influenced by factors like user behavior and advertising, which could affect the types of videos that are seen and shared.

## Conclusions

The present study highlights the significant changes in people's emotions, behaviors, and precautions toward COVID-19 during different periods of the pandemic. By analyzing the titles of YouTube videos posted during two different periods, we have identified a shift from a general lack of understanding and fear about the virus to a more nuanced approach to prevention and management. This shift is characterized by a focus on vaccination and travel-related precautions in the second group of videos, in contrast to the emphasis on basic prevention measures in the first group. These findings can inform public health policies and interventions aimed at mitigating the impact of COVID-19 and other pandemics. Furthermore, this study highlights the potential of social media platforms, such as YouTube, as a tool for monitoring and understanding public responses to pandemics and other health crises. As the COVID-19 pandemic continues to evolve, continued analysis of public responses and behaviors can help inform effective public health interventions and policies.

## References

[1] Wang C, Pan R, Wan X (2020). A longitudinal study on the mental health of general population during the COVID-19 epidemic in China. Brain Behav Immun.

[2] Alkhamees AA, Alrashed SA, Alzunaydi AA, Almohimeed AS, Aljohani MS (2020). The psychological impact of COVID-19 pandemic on the general population of Saudi Arabia. Compr Psychiatry.

[3] Basch CH, Hillyer GC, Meleo-Erwin ZC, Jaime C, Mohlman J, Basch CE (2020). Preventive behaviors conveyed on YouTube to mitigate transmission of COVID-19: cross-sectional study. JMIR Public Health Surveill.

[4] Basch CE, Basch CH, Hillyer GC, Jaime C (2020). The role of YouTube and the entertainment industry in saving lives by educating and mobilizing the public to adopt behaviors for community mitigation of COVID-19: successive sampling design study. JMIR Public Health Surveill.

[5] Dryhurst S, Schneider CR, Kerr J (2020). Risk perceptions of COVID-19 around the world. J Risk Res.

[6] Abu EK, Oloruntoba R, Osuagwu UL (2021). Risk perception of COVID-19 among sub-Sahara Africans: a web-based comparative survey of local and diaspora residents. BMC Public Health.

[7] Gozzi N, Tizzani M, Starnini M, Ciulla F, Paolotti D, Panisson A, Perra N (2020). Collective response to the media coverage of COVID-19 pandemic on Reddit and Wikipedia. J Med Internet Res.

[8] Pobiruchin M, Zowalla R, Wiesner M (2020). Temporal and location variations, and link categories for the dissemination of COVID-19-related information on Twitter during the SARS-CoV-2 outbreak in Europe: infoveillance study. J Med Internet Res.

[9] Holmes EA, O'Connor RC, Perry VH (2020). Multidisciplinary research priorities for the COVID-19 pandemic: a call for action for mental health science. Lancet Psychiatry.

[10] Chong TW, Curran E, Ames D, Lautenschlager NT, Castle DJ (2020). Mental health of older adults during the COVID-19 pandemic: lessons from history to guide our future. Int Psychogeriatr.

[11] Vasileiou E, Simpson CR, Shi T (2021). Interim findings from first-dose mass COVID-19 vaccination roll-out and COVID-19 hospital admissions in Scotland: a national prospective cohort study. Lancet.

[12] (2023). Centers for Disease Control and Prevention. Vaccines for COVID-19. https://www.cdc.gov/coronavirus/2019-ncov/vaccines/index.html.

[13] (2023). World Tourism Organization. Tourism and COVID-19. https://www.unwto.org/tourism-covid-19.

[14] (2023). European Centre for Disease Prevention and Control. COVID-19 and travel. https://www.ecdc.europa.eu/en/covid-19-and-travel.

[15] Thackeray R, Neiger BL, Smith AK, Van Wagenen SB (2012). Adoption and use of social media among public health departments. BMC Public Health.

